# Electrodynamics of Superconductors: From Lorentz to Galilei at Zero Temperature

**DOI:** 10.3390/e26010069

**Published:** 2024-01-12

**Authors:** Luca Salasnich

**Affiliations:** 1 Dipartimento di Fisica e Astronomia “Galileo Galilei” and Padua QTech Center, Universita di Padova, Via Marzolo 8, 35131 Padova, Italy; luca.salasnich@unipd.it; 2 Istituto Nazionale di Fisica Nucleare, Sezione di Padova, Via Marzolo 8, 35131 Padova, Italy; 3 Istituto Nazionale di Ottica del Consiglio Nazionale Delle Ricerche, Via Nello Carrara 2, 50127 Sesto Fiorentino, Italy

**Keywords:** superconductors, electrodynamics, zero temperature, zero entropy, nonrelativistic limit, penetration length, Bogoliubov spectrum, plasma frequency

## Abstract

We discuss the derivation of the electrodynamics of superconductors coupled to the electromagnetic field from a Lorentz-invariant bosonic model of Cooper pairs. Our results are obtained at zero temperature where, according to the third law of thermodynamics, the entropy of the system is zero. In the nonrelativistic limit, we obtain a Galilei-invariant superconducting system, which differs with respect to the familiar Schrödinger-like one. From this point of view, there are similarities with the Pauli equation of fermions, which is derived from the Dirac equation in the nonrelativistic limit and has a spin-magnetic field term in contrast with the Schrödinger equation. One of the peculiar effects of our model is the decay of a static electric field inside a superconductor exactly with the London penetration length. In addition, our theory predicts a modified D’Alembert equation for the massive electromagnetic field also in the case of nonrelativistic superconducting matter. We emphasize the role of the Nambu–Goldstone phase field, which is crucial to obtain the collective modes of the superconducting matter field. In the special case of a nonrelativistic neutral superfluid, we find a gapless Bogoliubov-like spectrum, while for the charged superfluid we obtain a dispersion relation that is gapped by the plasma frequency.

## 1. Introduction

There is a renewed interest in the phenomenological description of the superconductive electrodynamics taking explicitly into account relativistic effects [1,2,3,4,5] or the crucial role of the Nambu–Goldstone phase field [6,7] writing low-frequency and long-wavelength Lagrangians for neutral and charged fermionic superfluids [8,9,10,11,12,13,14,15,16,17,18,19]. Quite surprisingly, in the relativistic models of Refs. [1,2,3,4,5], the nonrelativistic limit of the relativistic matter field was not considered, somehow forgetting that the electrons, and the Cooper pairs, move at nonrelativistic velocities in the experimentally measured superconducting materials on earth [20,21,22,23].

In this paper, we fill this gap by investigating the nonrelativistic limit of a relativistic phenomenological model of bosonic Cooper pairs minimally coupled to the electromagnetic field. Quite remarkably, from the initial Lorentz-invariant setting, we obtain a Galilei-invariant theory for the superconducting matter field, which contains a crucial electromagnetic coupling term that is absent in the standard minimally coupled nonrelativistic Schrödinger field. This is exactly the analog of the coupling between the spin and the magnetic field one finds in the Pauli equation, which can be derived from the Dirac equation in the nonrelativistic limit [24]. By using our improved nonrelativistic formulation of the charged matter field, and explicitly taking into account the role of the Nambu–Goldstone phase field, working at zero temperature, where the entropy of the system is also zero, we predict effects that should be measurable at very low temperatures close to the absolute zero. Some of them have been previously suggested [1,2,3,4,5] but only assuming a quite unphysical relativistic matter field inside the superconductor. In particular, we suggest the decay of a static electric field inside a superconductor exactly with the London penetration depth. In addition, we obtain a modified D’Alembert equation for the massive electromagnetic waves inside the nonrelativistic superconducting matter. We finally derive a gapped spectrum for the density oscillations of the charged superfluid made of Cooper pairs. It is important to stress that many classical well-known experimental and theoretical results of superconductivity [20,21,22,23], such as the spontaneous symmetry breaking of gauge invariance, the London penetration depth of the magnetic field, and the collective modes of neutral superfluids, are fully recovered by our formalism.

## 2. Relativistic Cooper Pairs and Minimal Coupling

We assume that, at zero temperature, the Cooper pairs in a superconductor are described by a relativistic Klein–Gordon [25,26] complex scalar field φ(r,t) with Lagrangian density

(1) $$L_{0}=\frac{\hbar^{2}}{2mc^{2}}|\partial_{t}{\varphi |}^{2}-\frac{mc^{2}}{2}{\left|\phi \right|}^{2}-\frac{\hbar^{2}}{2m}{\left|\nabla \varphi \right|}^{2}-{E(|\varphi |}^{2})\;,$$

where $m=2m_{e}$ is the mass of a Cooper pair with $m_{e}$ the electron mass, *ℏ* is the reduced Planck constant, and *c* is the speed of light in vacuum. Here, $E(|\varphi {|}^{2})$ is the bulk internal energy of the system. The Lagrangian (1) is invariant with respect to Lorentz transformations. A similar model was developed by Govaerts, Bertrand, and Stenuit [1] and by Grigorishin [5]. However, in Refs. [1,5], the system is supposed to be close to the critical temperature $T_{c}$, with the temperature-dependent internal energy $E(|\varphi {|}^{2})$ given by the familiar quadratic–quartic Mexican hat potential. Here, instead, we work at zero temperature and, contrary to the previous papers, we want to emphasize the emerging properties in the nonrelativistic limit, where we obtain Maxwell–Proca equations [1,3,5] for the electromagnetic field coupled to the nonrelativistic superconducting matter. Quite remarkably, we find that the coupling between the electromagnetic field and the nonrelativistic matter contains a term that is absent by applying the minimal coupling to the electromagnetic field directly into a Schrödinger Lagrangian density. Recall that in the nonrelativistic limit from the Dirac equation of fermions, one obtains the Pauli equation (which has the spin) and not the Schrödinger equation (which does not have the spin) [24]. What is found here is the bosonic analog of that phenomenon.

The Cooper pair has the electric charge q=−2e with e>0 the modulus of the electron charge. The coupling with the electromagnetic field is obtained with the minimal substitution

(2) $$\partial_{t}\to \partial_{t}+i\frac{q}{\hbar }\Phi$$

(3) $$\nabla \to \nabla -i\frac{q}{\hbar }A$$

where Φ(r,t) is the electromagnetic scalar potential and A(r,t) is the electromagnetic vector potential, such that

(4) $$\begin{array}{c} E=-\nabla \Phi -\partial_{t}A \end{array}$$

(5) $$\begin{array}{c} B=\nabla \wedge A\;\;\;\;\;\;\;\;\;\; \end{array}$$

with E(r,t) the electric field and B(r,t) the magnetic field.

In this way, the total Lagrangian density $L_{\mathrm{tot}}$ of the system is given by using

(6) $$L_{\mathrm{tot}}=L_{\mathrm{shift}}+L_{\mathrm{em}}\;,$$

where

$$L_{\mathrm{shift}}=\frac{\hbar^{2}}{2mc^{2}}|\left(\partial_{t}+i\frac{q}{\hbar }\Phi \right){\varphi |}^{2}-\frac{\hbar^{2}}{2m}{\left|\left(\nabla -i\frac{q}{\hbar }A\right)\varphi \right|}^{2}\;\;\;\;$$

$$-{E(|\varphi |}^{2})-\frac{mc^{2}}{2}{\left|\phi \right|}^{2}\;\;\;\;\;\;\;\;\;\;\;\;\;\;\;\;\;\;\;\;\;\;\;\;\;\;\;\;\;\;\;\;\;\;\;\;\;\;\;\;\;\;\;\;\;\;\;\;\;\;\;\;\;\;\;$$

$$\;\;\;\;\;\;\;\;\;\;=L_{0}-\frac{mc^{2}}{2}{\left|\phi \right|}^{2}+\frac{q^{2}}{2mc^{2}}{\left|\varphi \right|}^{2}\Phi^{2}-\frac{iq\hbar }{2mc^{2}}\left(\varphi^{*}\partial_{t}\varphi -\varphi \partial_{t}\varphi^{*}\right)\Phi$$

(7) $$\;\;\;\;\;\;-\frac{q^{2}}{2m}{\left|\varphi \right|}^{2}A^{2}+\frac{iq\hbar }{2m}\left(\varphi^{*}\nabla \varphi -\varphi \nabla \varphi^{*}\right)\cdot A\;\;\;\;\;\;\;\;\;\;\;\;$$

is the shifted Lagrangian density of relativistic Cooper pairs and

(8) $$L_{\mathrm{em}}=\frac{\epsilon_{0}}{2}E^{2}-\frac{1}{2\mu_{0}}B^{2}$$

is the Lagrangian density of the free electromagnetic field, with $\epsilon_{0}$ the dielectric constant in the vacuum and $\mu_{0}$ the paramagnetic constant in the vacuum. Remember that $c=1/\sqrt{\epsilon_{0}\mu_{0}}$. The Lagrangian (6) is invariant with respect to the local U(1) gauge transformation $\varphi \left(r,t\right)\to \varphi \left(r,t\right)\;e^{i\alpha (r,t)}$.

As is well known, the total Lagrangian density (6) can be used to develop a zero-temperature quantum field theory by introducing the real-time partition function

(9) $$Z=\int D\left[\varphi ,\Phi ,A\right]\;e^{\frac{i}{\hbar }\int dt\;d^{3}r\;L_{\mathrm{tot}}}$$

of the system, within a functional integral formalism [9,15]. The theory can also be extended at finite temperature by performing a Wick rotation from real to imaginary time. However, in this paper, we do not consider the finite-temperature effects of the entropy-dependent normal component of the charged superfluid. The quantum expectation value of the scalar field φ(r,t) is defined as

(10) $$\langle \varphi \rangle =\frac{1}{Z}\int D\left[\varphi ,\Phi ,A\right]\;\varphi \;e^{\frac{i}{\hbar }\int dt\;d^{3}r\;L_{\mathrm{tot}}}\;.$$

The transition to the superconducting state is the breaking of the U(1) local gauge invariance, namely, 〈φ(r,t)〉≠0 [9,15]. In this paper, we work within the saddle-point approximation, where

(11) $$Z≃e^{\frac{i}{\hbar }\int dt\;d^{3}r\;L_{\mathrm{tot}}}$$

and the fields are the ones that extremize the action functional

(12) $$S_{\mathrm{tot}}=\int dt\;d^{3}r\;L_{\mathrm{tot}}\;.$$

In this mean-field framework, 〈φ(r,t)〉=φ(r,t) and quantum fluctuations are not taken into account.

## 3. From Lorentz to Galilei

The standard way to obtain a Galilei-invariant Schrödinger matter field ψ(r,t) from the Lorentz-invariant Klein–Gordon field φ(r,t) is to set

(13) $$\varphi \left(r,t\right)=\psi \left(r,t\right)\;e^{-imc^{2}t/\hbar }\;.$$

Inserting this ansatz into (1) and (7), we find

$$L_{0}\;=\frac{\hbar^{2}}{2mc^{2}}{\left|\partial_{t}\psi \right|}^{2}+\frac{i\hbar }{2}\left(\psi^{*}\partial_{t}\psi -\psi \partial_{t}\psi^{*}\right)$$

(14) $$-\frac{\hbar^{2}}{2m}{\left|\nabla \psi \right|}^{2}-{E(|\psi |}^{2})\;\;\;\;\;\;\;\;\;\;\;\;\;\;\;\;\;\;\;\;\;\;\;\;\;\;\;\;\;\;\;\;\;\;\;\;\;$$

and

$$L_{\mathrm{shift}}=L_{0}+\frac{q^{2}}{2mc^{2}}{\left|\psi \right|}^{2}\Phi^{2}-q{\left|\psi \right|}^{2}\Phi \;\;\;\;\;\;\;\;\;\;$$

$$\;\;\;\;\;\;\;\;\;\;\;-\frac{iq\hbar }{2mc^{2}}\left(\psi^{*}\partial_{t}\psi -\psi \partial_{t}\psi^{*}\right)\Phi -\frac{q^{2}}{2m}{\left|\psi \right|}^{2}A^{2}$$

(15) $$\;\;\;\;\;\;\;\;\;+\frac{iq\hbar }{2m}\left(\psi^{*}\nabla \psi -\psi \nabla \psi^{*}\right)\cdot A\;\;\;\;\;\;\;\;\;\;\;\;\;\;\;\;$$

At this point, the matter Lagrangian density is still Lorentz-invariant. However, under the assumption

(16) $$\frac{\hbar^{2}}{2mc^{2}}{\left|\partial_{t}\psi \right|}^{2}\ll \frac{i\hbar }{2}\left(\psi^{*}\partial_{t}\psi -\psi \partial_{t}\psi^{*}\right)$$

we obtain the approximated nonrelativistic Galilei-invariant Lagrangians

(17) $${\tilde{L}}_{0}=\frac{i\hbar }{2}\left(\psi^{*}\partial_{t}\psi -\psi \partial_{t}\psi^{*}\right)-\frac{\hbar^{2}}{2m}{\left|\nabla \psi \right|}^{2}-{E(|\psi |}^{2})$$

and

$${\tilde{L}}_{\mathrm{shift}}={\tilde{L}}_{0}+\frac{q^{2}}{2mc^{2}}{\left|\psi \right|}^{2}\Phi^{2}-{q|\psi |}^{2}\Phi -\frac{q^{2}}{2m}{\left|\psi \right|}^{2}A^{2}$$

(18) $$\;+\frac{iq\hbar }{2m}\left(\psi^{*}\nabla \psi -\psi \nabla \psi^{*}\right)\cdot A\;.\;\;\;\;\;\;\;\;\;\;\;\;\;\;\;\;\;$$

As expected, (17) is the Lagrangian density of a complex Schrödinger field ψ(r,t). Instead, quite remarkably, Equation (18) contains the crucial term $q^{2}\Phi^{2}{\left|\psi \right|}^{2}/\left(2mc^{2}\right)=\epsilon_{0}\mu_{0}q^{2}\Phi^{2}{\left|\psi \right|}^{2}/\left(2m\right)$. This term is absent by applying the minimal coupling to the electromagnetic field directly into a Schrödinger Lagrangian density. To better emphasize this relevant result, let us write the Euler–Lagrange equation of (18) with respect to $\psi^{*}\left(r,t\right)$, which is given by the following nonlinear Schrödinger equation

$$i\hbar \left(\partial_{t}+i\frac{q}{\hbar }\Phi \right)\psi = [-\frac{\hbar^{2}}{2m}{\left(\nabla -i\frac{q}{\hbar }A\right)}^{2}+\mu (|\psi^{2}|)]\psi$$

(19) $$\;\;-\frac{q^{2}}{mc^{2}}\Phi^{2}\;\psi \;,\;\;\;\;\;\;\;\;\;\;\;\;$$

where

(20) $${\mu (|\psi |}^{2})=\frac{\partial E}{{\partial |\psi |}^{2}}{\left(|\psi \right|}^{2})\;$$

is the chemical potential of the bulk system as a function of the local number density ${\left|\psi \right|}^{2}$. In Equation (19), it is the last term, which makes the nonrelativistic limit of the Klein–Gordon equation coupled to the electromagnetic field not equivalent to the fully nonrelativistic Schrödinger equation coupled to the electromagnetic field. This phenomenon is the analog of the Dirac equation coupled to the electromagnetic field: in the nonrelativistic limit from the Dirac equation, one obtains the Pauli equation (which has the spin) and not the Schrödinger equation (which does not have the spin) [24]. Note that the term $q^{2}\Phi^{2}/\left(mc^{2}\right)=\epsilon_{0}\mu_{0}\Phi^{2}/m$ can be discarded only if $\left|q\Phi \right|\ll mc^{2}$, but in nonrelativistic superconductors, this is not the case.

## 4. Density-Phase Lagrangian

The Schrödinger field ψ(r,t) of Equations (17) and (18) is the order parameter of nonrelativistic Cooper pairs. We now set

(21) $$\psi =\sqrt{n_{s}}\;e^{i\theta }\;,$$

where $n_{s}\left(r,t\right)$ is the number density of Cooper pairs of mass *m* and electric charge *q*, while θ(r,t) is the Nambu–Goldstone phase field [6,7].

Several authors [8,9,10,11,12,13,14,15,19] adopted the idea of writing a low-frequency and long-wavelength Lagrangian density L of a nonrelativistic superfluid in terms of θ(r,t). In all these approaches, the local superfluid velocity field $v_{s}\left(r,t\right)$ is related to θ(r,t) via the fundamental relationship

(22) $$v_{s}=\frac{\hbar }{m}\nabla \theta \;.$$

This equation ensures that the fluid is irrotational, i.e., $\nabla \wedge v_{s}=0$, apart from a set of zero-measure quantized vortices. Indeed, in the presence of a quantized vortex with integer quantum number κ, around it the circulation of the superfluid velocity is such that [27,28]

(23) $$\oint v_{s}\cdot dr=\frac{\hbar }{m}\oint \nabla \theta \cdot dr=\frac{\hbar }{m}\int_{0}^{2\pi \kappa }d\theta =\frac{2\pi \hbar }{m}\kappa \;.$$

Inserting Equation (21) into Equations (17) and (18), we obtain the following total Lagrangian density

(24) $$L_{\mathrm{tot}}={\tilde{L}}_{0}+L_{\mathrm{em}}+{\tilde{L}}_{I}\;,$$

where

(25) $${\tilde{L}}_{0}=-n_{s}\;\hbar \partial_{t}\theta -n_{s}\;\frac{\hbar^{2}}{2m}{\left(\nabla \theta \right)}^{2}-\frac{\hbar^{2}}{8m}\frac{{\left(\nabla n_{s}\right)}^{2}}{n_{s}}-E\left(n_{s}\right)$$

is our nonrelativistic density-phase Lagrangian, $L_{\mathrm{em}}$ is the Lagrangian of the free electromagnetic field, given by Equation (8), and

(26) $${\tilde{L}}_{I}=n_{s}\;\epsilon_{0}\mu_{0}\frac{q^{2}\Phi^{2}}{2m}-n_{s}\;q\Phi -n_{s}\;\frac{q^{2}A^{2}}{2m}+n_{s}\;q\;\frac{\hbar }{m}\nabla \theta \cdot A\;$$

is the Lagrangian of the interaction between the Cooper pairs and the electromagnetic field.

We observe that in Equation (25), the von Weizsäcker-like term [29] appears

(27) $${\tilde{L}}_{0,W}=-\frac{\hbar^{2}}{8m}\frac{{\left(\nabla n_{s}\right)}^{2}}{n_{s}}$$

which takes into account the energy cost due to variations in the superfluid density. As we will see, this term modifies the dispersion relation of the collective modes of both neutral and charged superfluids. In a very recent paper [19], it has been suggested that Equation (27) is crucial to obtain a negative electrohydrostatic pressure between superconducting bodies at zero temperature.

### 4.1. Including the Ion Background

In the ground state of a superconductor, there is a compensation between the negative electric charge density $\rho_{s}=qn_{s}=-2en_{s}$ of the Cooper pairs and the positive electric charge density $\rho_{\mathrm{bg}}=-q{\bar{n}}_{\mathrm{bg}}=2e{\bar{n}}_{\mathrm{bg}}$ of the background of ions with an average number density ${\bar{n}}_{\mathrm{ions}}$. Taking into account this fact, similarly to the Jellium model of a metallic conductor [20], the full Lagrangian of our model is given by

(28) $$L_{\mathrm{full}}=L_{\mathrm{tot}}+L_{\mathrm{bg}}\;,$$

where

(29) $$L_{\mathrm{bg}}={\bar{n}}_{\mathrm{bg}}\;q\Phi \;.$$

Notice that we are assuming that the number density ${\bar{n}}_{\mathrm{bg}}$ of the ion background is space-time-independent.

For the sake of clarity, we stress that in this nonrelativistic framework, it is the Lagrangian density (28) that must be used to obtain the path-integral partition function

(30) $$Z_{\mathrm{full}}=\int D\left[n_{s},\theta ,\Phi ,A\right]\;e^{\frac{i}{\hbar }\int dt\;d^{3}r\;L_{\mathrm{full}}}\;,$$

where the functional integration is performed with respect to the local number density $n_{s}\left(r,t\right)$ of the Cooper pairs, the Nambu–Goldstone field θ(r,t), and the electromagnetic potentials Φ(r,t) and A(r,t).

### 4.2. Charge Density and Current Density

It is impossible to calculate analytically Equation (30). However, the saddle-point (mean-field) solution is the set of Euler–Lagrange equations that are obtained by extremizing the action functional

(31) $$S_{\mathrm{full}}=\int dt\;d^{3}r\;L_{\mathrm{full}}\;.$$

The Euler–Lagrange equations of the full Lagrangian (28) with respect to the scalar potential Φ(r,t) and the vector potential A(r,t) are nothing else than the Maxwell equations

(32) $$\nabla \cdot E=\frac{\rho }{\epsilon_{0}}$$

(33) ∇·B=0

(34) $$\nabla \wedge E=-\partial_{t}B$$

(35) $$\nabla \wedge B=\mu_{0}\;j+\epsilon_{0}\mu_{0}\;\partial_{t}E$$

where the expressions of the local charge density ρ(r,t) and the local current density j(r,t) are given by

(36) $$\rho =-\frac{\partial \left({\tilde{L}}_{I}+L_{\mathrm{bg}}\right)}{\partial \Phi }$$

(37) $$j=\frac{\partial {\tilde{L}}_{I}}{\partial A}\;\;\;\;\;\;\;\;\;\;\;\;\;\;\;$$

Thus, one obtains

(38) $$\rho =q\;n_{s}-q\;{\bar{n}}_{\mathrm{bg}}-\epsilon_{0}\;\frac{q^{2}n_{s}\mu_{0}}{m}\;\Phi$$

(39) $$j=q\;n_{s}v_{s}-\frac{1}{\mu_{0}}\frac{q^{2}n_{s}\mu_{0}}{m}\;A\;,\;\;\;\;\;\;$$

where the first term

(40) $$\rho_{s}=q\;n_{s}$$

in Equation (38) is the electric charge density of the Cooper pairs, the second term is the electric charge density of the ion background, and the third term

(41) $$\rho_{I}=-\frac{\epsilon_{0}\mu_{0}q^{2}n_{s}}{m}\;\Phi$$

in Equation (38) is the interaction charge density due to the coupling between the Cooper pairs and the electromagnetic scalar potential Φ. Instead, the first term

(42) $$j_{s}=q\;n_{s}v_{s}$$

in Equation (39) is the electric current density of Cooper pairs, which contains the superfluid velocity $v_{s}$ defined in Equation (22). The second term

(43) $$j_{I}=-\frac{q^{2}n_{s}}{m}\;A$$

in Equation (39) is nothing else than the London current [30] due to the interaction between the Cooper pairs and the electromagnetic vector potential A. In 1935, Fritz and Heinz London [30] introduced a nonrelativistic model where the interaction density $\rho_{I}$ was included in the total electric charge density ρ. However, due to the lack of experimental evidence [31], subsequently Fritz London discarded this term in his book [32]. In recent years, it has been suggested by Hirsch within an alternative model [2,4] that, at very low temperatures, the interaction density $\rho_{I}$ could be effective and measurable.

We underline that, as is well known, manipulating Equations (32) and (35) one finds the continuity equation for the electric charge density ρ and the electric current density j, namely [33],

(44) $$\partial_{t}\rho +\nabla \cdot j=0\;.$$

This result will be used later in combination with a similar, but not equal, continuity equation for the superconductive charge density $\rho_{s}$ of Cooper pairs and the electric current density j.

Equations (32) and (33) equipped with Equations (38) and (39) are nothing else than the Maxwell–Proca equations for the electrodynamics of the superconductors previously discussed in Refs. [1,3]. However, in Ref. [1], the Maxwell–Proca equations are obtained from a finite-temperature relativistic model, while in Ref. [3] these equations are heuristically introduced without a derivation. Here, we will analyze the consequences of the Maxwell–Proca equations for superconductors at zero temperature, where the normal density is absent. Moreover, we will investigate the collective modes of charged superfluid.

Deep inside a superconductor, both the magnetic field B and electric field E are zero [21]. As a consequence, from our Equations (32) and (38), it follows that for the ground state, characterized by a uniform and constant number density ${\bar{n}}_{s}$ of Cooper pairs and a vanishing electromagnetic potential Φ=0, the total electric charge density ρ is zero, namely,

(45) $$0=q\left({\bar{n}}_{s}-{\bar{n}}_{\mathrm{bg}}\right)\;,$$

and consequently ${\bar{n}}_{s}={\bar{n}}_{\mathrm{bg}}$. As previously discussed, the ion background neutralizes the system.

### 4.3. London Penetration Depth for the Static Magnetic Field

As discussed above, Equation (39) was obtained for the first time by the London brothers [30] and it gives rise to the expulsion of a magnetic field from a superconductor (Meissner–Ochsenfeld effect) [34].

In a static configuration with a zero superfluid velocity $v_{s}$ and in the absence of the electric field, i.e., E=0, the curl of Equation (35) gives

(46) $$-\nabla^{2}B=\mu_{0}\;\nabla \wedge \left(-\frac{q^{2}n_{s}}{m}A\right)\;,$$

taking into account that

(47) $$\nabla \wedge \left(\nabla \wedge B\right)=-\nabla^{2}B+\nabla \left(\nabla \cdot B\right)=-\nabla^{2}B$$

due to the Gauss law, Equation (33). Assuming that the local density $n_{s}\left(r\right)$ is uniform, i.e., $n_{s}\left(r\right)={\bar{n}}_{s}$, by using Equation (5), we obtain

(48) $$\nabla^{2}B=\frac{q^{2}{\bar{n}}_{s}\mu_{0}}{m}B\;.$$

Choosing the magnetic field as B=B(x)u, with u a unit vector, the previous equation can be written as

(49) $$\frac{\partial^{2}}{\partial x^{2}}B=\frac{q^{2}{\bar{n}}_{s}\mu_{0}}{m}B$$

which has the following physically relevant solution for a superconducting slab defined in the region x≥0:

(50) $$B\left(x\right)=B\left(0\right)\;e^{-x/\lambda_{L}}\;,$$

where

(51) $$\lambda_{L}=\sqrt{\frac{m}{q^{2}{\bar{n}}_{s}\mu_{0}}}$$

is the so-called London penetration depth, which is typically around 100 nm [21]. The meaning of Equation (50) is that inside a superconductor, the static magnetic field decays exponentially. This is the Meissner–Ochsenfeld effect: the expulsion of a magnetic field from a superconductor, experimentally observed for the first time in 1933 [34].

### 4.4. London Penetration Depth for the Static Electric Field

It is well know that normal metals screen an external electric field E, which can penetrate at most a few angströms (Thomas–Fermi screening length) [20]. For superconducting materials, our Equations (32), (33), (38), and (39) suggest that the electric field E exponentially decays inside a zero-temperature superconductor with the much larger London penetration depth $\lambda_{L}$. Let us show how to derive this relevant result within our theoretical framework.

In a static configuration, in the absence of the magnetic field, i.e., **B** = **0**, and assuming a uniform number density, the gradient of Equation (32), with Equations (38) and (51), gives

(52) $$\nabla^{2}E=-\frac{1}{\lambda_{L}^{2}}\nabla \Phi \;,$$

taking into account that

(53) $$\nabla \left(\nabla \cdot E\right)=\nabla^{2}E-\nabla \wedge \left(\nabla \wedge E\right)=\nabla^{2}E\;.$$

Notice that to obtain Equation (52), it is crucial to assume a uniform background $n_{\mathrm{bg}}$. In addition, due to Equation (4), we find

(54) $$\nabla^{2}E=\frac{1}{\lambda_{L}^{2}}E\;.$$

Choosing E=E(x)u, with u a unit vector, the previous equation can be written as

(55) $$\frac{\partial^{2}}{\partial x^{2}}E=\frac{1}{\lambda_{L}^{2}}E$$

which has the following physically relevant solution for a superconducting slab defined in the region x≥0:

(56) $$E\left(x\right)=E\left(0\right)\;e^{-x/\lambda_{L}}\;.$$

The meaning of Equation (56) is that inside a zero-temperature superconductor, the static electric field decays exponentially with a characteristic decay length that is exactly the London penetration depth $\lambda_{L}$.

### 4.5. Modified D’Alembert Equation for Electromagnetic Waves

We investigate what happens to an electromagnetic wave when it is suddenly applied to a superconductor in its ground state. In full generality, from the Maxwell Equations (32)–(35), one obtains the inhomogeneous wave equations [33]

(57) $$\left(\frac{1}{c^{2}}\frac{\partial^{2}}{\partial t^{2}}-\nabla^{2}\right)E=-\frac{1}{\epsilon_{0}}\nabla \rho -\mu_{0}\partial_{t}j$$

(58) $$\left(\frac{1}{c^{2}}\frac{\partial^{2}}{\partial t^{2}}-\nabla^{2}\right)B=\mu_{0}\nabla \wedge j\;.\;\;\;\;\;\;\;\;\;\;\;$$

Under the assumption that the local number density of Cooper pairs remains approximately constant and uniform, i.e., $n_{s}\left(r,t\right)≃{\bar{n}}_{s}$, and with a zero superfluid velocity, i.e., $v_{s}\left(r,t\right)≃0$, after remembering Equations (4), (5), (36), and (37), from Equations (57) and (58), we obtain

(59) $$\left(\frac{1}{c^{2}}\frac{\partial^{2}}{\partial t^{2}}-\nabla^{2}+\frac{1}{\lambda_{L}^{2}}\right)E=0$$

(60) $$\left(\frac{1}{c^{2}}\frac{\partial^{2}}{\partial t^{2}}-\nabla^{2}+\frac{1}{\lambda_{L}^{2}}\right)B=0$$

that are the modified D’Alembert equation for the electromagnetic waves inside the superconductor with $\lambda_{L}$ being the London penetration depth of Equation (51).

The Fourier transform of Equations (59) and (60) in the frequency–wavevector domain (ω,k) gives the dispersion relation

(61) $$\omega =\sqrt{\omega_{p}^{2}+c^{2}k^{2}}\;,$$

where

(62) $$\omega_{p}=\frac{c}{\lambda_{L}}=\sqrt{\frac{q^{2}{\bar{n}}_{s}}{m\epsilon_{0}}}$$

is the plasma frequency [20]. Thus, the photon spectrum becomes gapped or, in other words, the photon acquires a mass. This is nothing else than the Anderson–Higgs mechanism [35,36,37], which survives in our model also in the context of nonrelativistic superconducting matter. Notice that Equation (61) appears also in Refs. [2,5]. As discussed in Ref. [5], the dispersion relation (61) can be also written as

(63) $$k=\frac{1}{c}\sqrt{\omega^{2}-\omega_{p}^{2}}=\sqrt{\frac{\omega^{2}}{c^{2}}-\frac{1}{\lambda_{L}^{2}}}\;.$$

Consequently, the electromagnetic plane wave that is proportional to $e^{i(k\cdot r-\omega t)}$ propagates without dissipation inside the superconductor for $\omega >\omega_{p}$. Instead, for $\omega <\omega_{p}$, the electromagnetic wave is damped as $e^{-u\cdot r/\lambda_{\omega }}\;e^{-i\omega t}$ in the interior of the superconductor, where $k=iu/\lambda_{\omega }$ with u a unit vector and

(64) $$\lambda_{\omega }=\frac{\lambda_{L}}{\sqrt{1-{\left(\frac{\omega }{\omega_{p}}\right)}^{2}}}$$

is the frequency-dependent penetration depth. Clearly, $\lambda_{\omega }\to \lambda_{L}$ as ω→0. Moreover, indicating with Δ(0) the energy gap of the Cooper pairs at zero temperature, for ω>2Δ(0)/ℏ, the charged superfluid becomes a normal charged fluid due to the breaking of the Cooper pairs.

## 5. Euler–Lagrange Equations of Superconductors

The Euler–Lagrange equation of the full Lagrangian (28) with respect to the Nambu–Goldstone field θ(r,t) reads as

(65) $$\partial_{t}n_{s}+\nabla \cdot \left(n_{s}v_{s}-\frac{qn_{s}}{m}A\right)=0\;.$$

This is nothing else than the continuity equation

(66) $$\partial_{t}\rho_{s}+\nabla \cdot j=0\;,$$

where the local superconducting charged density $\rho_{s}\left(r,t\right)$ is given by Equation (40) and the local charged current density j(r,t) is given by Equation (39). Comparing Equation (66) with Equation (44), it follows that

(67) $$\partial_{t}\left(\rho -\rho_{s}\right)=0\;,$$

i.e., the interaction charge density $\rho_{I}=\rho -\rho_{s}$ given by Equation (41) must be time-independent or, equivalently,

(68) $$\Phi \;\partial_{t}n_{s}=-n_{s}\;\partial_{t}\Phi \;.$$

Instead, the Euler–Lagrange equation for the local number density $n_{s}\left(r,t\right)$ leads to

$$\hbar \;\partial_{t}\theta +q\Phi -\frac{\epsilon_{0}\mu_{0}q^{2}}{2m}\Phi^{2}-\frac{q}{m}\nabla \theta \cdot A+\frac{q^{2}A^{2}}{2m}+\frac{\hbar^{2}}{2m}{\left(\nabla \theta \right)}^{2}$$

(69) $$\;\;+\frac{\partial E}{\partial n_{s}}-\frac{\hbar^{2}}{2m\sqrt{n_{s}}}\nabla^{2}\sqrt{n_{s}}=0\;.\;\;\;\;\;\;\;\;\;\;\;\;\;\;\;\;\;\;\;\;\;\;\;\;\;\;$$

By applying the gradient operator **∇** to Equation (69), one finds

$$m\partial_{t}v_{s}+\nabla [\frac{1}{2}mv_{s}^{2}+\mu \left(n_{s}\right)-\frac{\hbar^{2}}{2m\sqrt{n_{s}}}\nabla^{2}\sqrt{n_{s}}+q\Phi$$

(70) $$\;-\frac{\epsilon_{0}\mu_{0}q^{2}}{2m}\Phi^{2}-\frac{q}{\hbar }v_{s}\cdot A+\frac{q^{2}A^{2}}{2m}]=0\;,$$

where $\mu (n_{s})$, given by Equation (20), is the chemical potential of the bulk system as a function of the local number density $n_{s}\left(r,t\right)$.

### 5.1. Gapless Collective Modes of Neutral Superfluids

In the very special case of a neutral superfluid, i.e., if q=0, the previous equations become much simpler and it is quite easy to determine the collective modes of the zero-temperature neutral superfluid. We set

(71) $$n_{s}\left(r,t\right)={\bar{n}}_{s}+{\delta n}_{s}\left(r,t\right)$$

(72) $$v_{s}\left(r,t\right)=0+{\delta v}_{s}\left(r,t\right)$$

assuming that ${\delta n}_{s}\left(r,t\right)$ and ${\delta v}_{s}\left(r,t\right)$ are small perturbations with respect to the ground-state configuration with a uniform number density ${\bar{n}}_{s}$ and zero superfluid velocity.

Under the condition q=0, the linearized version of Equations (65) and (70) are then given by

(73) $$\;\;\;\;\;\;\;\;\;\;\;\;\;\;\;\;\;\;\;\;\;\;\partial_{t}{\delta n}_{s}+{\bar{n}}_{s}\nabla \cdot {\delta v}_{s}=0\;,$$

(74) $${\bar{n}}_{s}\partial_{t}{\delta v}_{s}+c_{s}^{2}\nabla {\delta n}_{s}-\frac{\hbar^{2}}{4m^{2}}\nabla \left(\nabla^{2}{\delta n}_{s}\right)=0\;,$$

where

(75) $$c_{s}=\sqrt{\frac{{\bar{n}}_{s}}{m}\frac{\partial \mu }{\partial n}\left({\bar{n}}_{s}\right)}$$

is the speed of sound. By applying the time derivative $\partial_{t}$ to Equation (73) and the divergence ∇· to Equation (74) and subtracting the two resulting equations, we find

(76) $$\left(\partial_{t}^{2}-c_{s}^{2}\nabla^{2}+\frac{\hbar^{2}}{4m^{2}}\nabla^{4}\right){\delta n}_{s}=0\;,$$

The Fourier transform of Equation (76) in the frequency–wavevector domain (ω,k) gives the dispersion relation

(77) $$\omega =\sqrt{c_{s}^{2}k^{2}+\frac{\hbar^{2}k^{4}}{4m^{2}}}\;.$$

This dispersion relation is a gapless Bogoliubov-like spectrum [38], which reduces to the phonon spectrum

(78) $$\omega =c_{s}k$$

at very low wavenumbers, while for large wavenumbers one finds

(79) $$\omega =\frac{\hbar k^{2}}{2m}$$

that is the single-particle spectrum of free massive particles. For the sake of completeness, we underline that Equation (77) is fully consistent with our previous results [16,17,18] for the collective modes of nonrelativistic neutral fermionic superfluids with the inclusion of the von Weizsäcker-like term, Equation (27).

### 5.2. Gapped Collective Modes of Charged Superfluids

We now analyze the collective modes of a zero-temperature superconductor. In this case q≠0 and, in addition to Equations (71) and (72), we must set

(80) Φ(r,t)=0+δΦ(r,t)

(81) A(r,t)=0+δA(r,t)

assuming that δΦ(r,t) and δA(r,t) are small perturbations with respect to the ground-state electromagnetic configuration of the zero scalar potential and zero vector potential.

Under the condition q≠0, the linearized versions of Equations (65) and (70) are then given by

(82) $$\partial_{t}\left({\delta n}_{s}\right)+{\bar{n}}_{s}\nabla \cdot {\delta v}_{s}-\frac{q{\bar{n}}_{s}}{m}\nabla \cdot \delta A=0\;,$$

$${\bar{n}}_{s}\partial_{t}\left({\delta v}_{s}\right)+c_{s}^{2}\nabla \left({\delta n}_{s}\right)-\frac{\hbar^{2}}{4m^{2}}\nabla \left(\nabla^{2}{\delta n}_{s}\right)$$

(83) $$+\frac{q{\bar{n}}_{s}}{m}\nabla \left(\delta \Phi \right)=0\;.$$

Similarly, the linearized version of the Maxwell Equations (32)–(35) reads

(84) $$\nabla \cdot \delta E=\frac{\delta \rho }{\epsilon_{0}}$$

(85) ∇·δB=0

(86) $$\nabla \wedge \delta E=\partial_{t}\left(\delta B\right)$$

(87) $$\nabla \wedge \delta B=\mu_{0}\;\delta j+\epsilon_{0}\mu_{0}\;\partial_{t}\left(\delta E\right)$$

where

(88) $$\delta E=-\nabla \left(\delta \Phi \right)-\partial_{t}\left(\delta A\right)$$

(89) δB=∇∧δA

and

(90) $$\delta \rho =q\;\delta n_{s}-\epsilon_{0}\frac{q^{2}{\bar{n}}_{s}\mu_{0}}{m}\;\delta \Phi \;\;\;\;\;\;\;\;\;\;\;\;\;\;\;\;\;\;\;\;\;\;\;\;\;\;$$

(91) $$\delta j=q\;{\bar{n}}_{s}\delta v_{s}-\frac{1}{\mu_{0}}\frac{q^{2}\mu_{0}}{m}A\;\delta n_{s}-\frac{1}{\mu_{0}}\frac{q^{2}{\bar{n}}_{s}\mu_{0}}{m}\;\delta A.$$

Needless to say, finding analytical solutions for the coupled equations from (82) to (91) seems not easy. However, we are able to obtain some interesting results. By applying the time derivative $\partial_{t}$ to Equation (82) and the divergence ∇· to Equation (83) and subtracting the two resulting equations, we find

(92) $$\left(\partial_{t}^{2}-c_{s}^{2}\nabla^{2}+\frac{\hbar^{2}}{4m^{2}}\nabla^{4}\right){\delta n}_{s}+\frac{q{\bar{n}}_{s}}{m}\nabla \cdot \delta E=0\;,$$

taking into account Equation (88). Then, from the first Maxwell Equation (84) and Equation (90), we obtain

(93) $$\nabla \cdot \delta E=\frac{\delta \rho }{\epsilon_{0}}=\frac{q}{\epsilon_{0}}\delta n_{s}+\frac{\delta \rho_{I}}{\epsilon_{0}}\;.$$

Remembering that $\rho_{I}=\rho -\rho_{s}$ is constant in time, as shown by Equation (67), by applying the operator $\partial_{t}$ to Equation (92), we obtain

(94) $$\left(\partial_{t}^{3}-c_{s}^{2}\partial_{t}\nabla^{2}+\frac{\hbar^{2}}{4m^{2}}\partial_{t}\nabla^{4}+\omega_{p}^{2}\partial_{t}\right){\delta n}_{s}=0\;,$$

which gives the dispersion relation ω=0 but also

(95) $$\omega =\sqrt{\omega_{p}^{2}+c_{s}^{2}k^{2}+\frac{\hbar^{2}k^{4}}{4m^{2}}}$$

that is a gapped generalization of Equation (77). As expected, the gap is exactly due to the plasma frequency $\omega_{p}$ of Equation (62).

## 6. Conclusions

We have analyzed several consequences of a time-dependent relativistic model of bosonic-charged Cooper pairs minimally coupled to the electromagnetic field. While our model shares similarities with other relativistic treatments of superconductivity [1,2,3,4,5], it differs in at least three important ways. First, our results have been obtained at zero temperature where, at least for clean superconductors, the normal component of the superconducting electrons is zero and a real-time description of the superconductive bosonic field is fully justified [39]. Second, we have explicitly discussed the derivation of the nonrelativistic model for the matter field from the relativistic one, emphasizing the crucial role of a term that couples the density of Cooper pairs with the electromagnetic scalar potential. This term can be also obtained [40] from the nonrelativistic low-frequency and long-wavelength Popov’s action [8,9] of a charged superfluid but only when performing a quantum-mechanical–functional integration with respect to the density field within the saddle-point approximation. Third, we have obtained the full set of equations for nonrelativistic charged superfluids coupled to the (relativistic) electromagnetic field in terms of the superfluid density and the superfluid velocity, which is directly related to the gradient of the Nambu–Goldstone phase field. In these equations, in addition to the previously discussed coupling term, there is a von Weizsäcker-like term [29], which takes into account the energy cost due to variations in the superfluid density and modifies the dispersion relation of the superfluid collective modes.

Our model supports the claim [2,4,5] that, very close to zero temperature, it should be possible to experimentally measure the decay of a static electric field inside a superconductor with a characteristic length that is the London penetration depth instead of the Thomas–Fermi screening length. A recent experimental attempt to measure this effect by using atomic force microscopy on a niobium sample was inconclusive due to limited accuracy [41]. We expect that near-future experiments could test also other zero-temperature predictions discussed in this paper: a gapped spectrum of the electromagnetic waves inside the superconductor and the gapped spectrum of the superconducting density oscillations. To achieve these goals, it is necessary to work at extremely low temperatures, where the normal component, containing the entropy and the viscosity of the system, is negligible. This is the main experimental problem that needs to be overcome.

## Data Availability

Data are contained within the article.
